# Response

**DOI:** 10.1007/s00392-023-02233-0

**Published:** 2023-06-22

**Authors:** David Grundmann, Niklas Schofer

**Affiliations:** Department of Cardiology, University Heart and Vascular Center Hamburg, University Medical Center Hamburg-Eppendorf, Martinistraße 52, 20246 Hamburg, Germany

Sirs,

Dear Professor Böhm, Dear Professor Frey,

In this letter you will find our response to Comment on Grundmann et al. “Prognostic impact and diagnostic value of invasively derived hemodynamic measures in patients with severe aortic stenosis undergoing TAVI”.

We thank for the interest in and the comment on our scientific study.

First, all data was derived from a secondary care setting.

Second, as stated in the manuscript, all of our findings should be interpreted as hypothesis-generating. Thus, no causal conclusions should be drawn on the sole basis of the presented retrospective data. However, we believe that our findings justify further (prospective) investigation regarding the diagnostic utility of TLVAo as one additional component of an multimodal approach for the assessment of AS severity. The same holds true for the putative prognostic value of ET and AT, which could be useful e.g. as an integrated part of a clinical risk score.

Sincerely yours

David Grundmann and Niklas Schofer.

